# Oxidative Stress Induces Monocyte Necrosis with Enrichment of Cell-Bound Albumin and Overexpression of Endoplasmic Reticulum and Mitochondrial Chaperones

**DOI:** 10.1371/journal.pone.0059610

**Published:** 2013-03-26

**Authors:** Haiping Tang, Enbing Tian, Chongdong Liu, Qingtao Wang, Haiteng Deng

**Affiliations:** 1 MOE Key Laboratory of Bioinformatics, School of Life Sciences, Tsinghua University, Beijing, China; 2 Beijing Chaoyang Hospital Affiliated to Capital Medical University, Beijing, China; Institut national de la santé et de la recherche médicale (INSERM), France

## Abstract

In the present study, monocytes were treated with 5-azacytidine (azacytidine), gossypol or hydrogen peroxide to induce cell death through oxidative stress. A shift from apoptotic to necrotic cell death occurred when monocytes were treated with 100 µM azacytidine for more than 12 hours. Necrotic monocytes exhibited characteristics, including enrichment of cell-bound albumin and up-regulation of endoplasmic reticulum (ER)- and mitochondrial-specific chaperones to protect mitochondrial integrity, which were not observed in other necrotic cells, including HUH-7, A2780, A549 and HOC1a. Our results show that the cell-bound albumin originates in the culture medium rather than from monocyte-derived hepatocytes, and that HSP60 is a potential binding partner of the cell-bound albumin. Proteomic analysis shows that HSP60 and protein disulfide isomerase are the most abundant up-regulated mitochondrial and ER-chaperones, and that both HSP60 and calreticulin are ubiquitinated in necrotic monocytes. In contrast, expression levels of the cytosolic chaperones HSP90 and HSP71 were down-regulated in the azacytidine-treated monocytes, concomitant with an increase in the levels of these chaperones in the cell culture medium. Collectively, our results demonstrates that chaperones from different organelles behave differently in necrotic monocytes, ER- and mitochondrial chaperones being retained and cytosolic and nuclear chaperones being released into the cell culture medium through the ruptured cell membrane. HSP60 may serve as a new target for development of *myeloid* leukemia treatment.

## Introduction

Necrosis is a type of cell death that lacks the characteristics of apoptosis and autophagy [1]–[5]. Over the last several years, it has been found that the occurrence and course of necrosis are programmed and tightly regulated. Extensive studies have shown that death ligands (*e.g.,* CD95L, TNF and TNF-related apoptosis-inducing ligand) induce caspase-independent necrotic-like cell death that relies on the activity of the death domain (DD)-containing kinase Rip1. Although the inductive mechanisms of necrosis are becoming increasingly clear, the execution of this process remains somewhat elusive. Necrosis is associated with specific cellular processes such as mitochondrial dysfunction, enhanced generation of reactive oxygen species, ATP depletion, proteolysis by calpains and cathepsins, and early plasma membrane rupture. One consequence of necrosis is the induction of immunogenic responses pursuant to the release of immunogens from necrotic cells [6]–[9]. *Basu* and colleagues reported that heat shock proteins (HSPs) including gp96, calreticulin, HSP90 and HSP72 were released into the culture supernatant in response to freeze thaw in necrotic cells, but not in apoptotic cells [10]–[11]. It was further shown that the released HSPs activated the NF-κB pathway, stimulated macrophages to secrete cytokines, induced the expression of co-stimulatory molecules, and enhanced antigen presentation in dendritic cells [12]–[17].

Necrosis of monocytes and macrophages has been well characterized. Exposure of THP-1 cells to aqueous peroxyl radical has been shown to result in glutathione loss followed by protein oxidation and caspase-3-independent cell death, suggesting that oxidative stress causes monocyte necrosis [18]. Moreover, inhibition of Rip1 and Rip3 activation by cIAP1 and cIAP2 limits macrophage necrosis [19]. In pathogen-induced monocyte/macrophage necrosis, NLRP3 plays a critical role in necrotic death triggered by Mycobacterium tuberculosis [20]. In addition, cathepsin has been identified as the downstream executor for necrosis: mutations in CIAS1 induced cathepsin B-dependent rapid cell death of human THP-1 monocytic cells [21]. Moreover, Legionella pneumophila has been shown to induce cathepsin B-dependent necrotic cell death through release of high mobility group box1 in macrophages [22]. It has also been demonstrated that the activities of cathepsin and HSP90 determine the balance between apoptotic and necrotic cell death pathways in caspase-compromised U937 cells [23]. In contrast, however, changes in protein expression in necrotic monocytes have not been systematically investigated, and proteomic analysis will provide important information for identification of the key proteins and for deciphering molecular events in monocyte necrosis.

In the present study, monocyte cell lines were treated with azacytidine, gossypol or hydrogen peroxide to induce cell necrosis through oxidative stress. Using proteomic analysis, we found that the necrotic monocytes exhibited enrichment of cell-bound albumin that originated in culture medium rather than from monocyte-derived hepatocytes. Oxidative stress also induced differential changes in chaperones from distinct organelles. HSP60 was overexpressed and ubiquitinated in necrotic monocytes while HSP71 and HSP90 were released into the cell culture medium.

## Materials and Methods

### Chemicals and Reagents

RPMI-1640 medium, phosphate-buffered saline (PBS) and fetal bovine serum were purchased from Wisent (Montreal, QC) and used without further purification. Dithiothreitol (DTT) was purchased from Merck (Whitehouse Station, NJ). Sequencing grade modified trypsin was purchased from Promega (Fitchburg, WI). Crystallized bovine serum albumin (BSA) was purchased from Amresco (Solon, OH). 5-azacytidine, iodoacetamide (IAA) and RNase A were purchased from Sigma (St Louis, MO). Dimethyl sulfoxide was purchased from Applichem (St Louis, MO). The Apoptosis & Necrosis Quantification Kit was purchased from Biotium, (Hayward, CA). Anti-albumin antibody was purchased from Abcam (Cambridge, MA). Anti-bovine serum albumin antibody was purchased from Invitrogen (Grand Island, NY). Anti-HSP90 and anti-HSP60 antibodies were purchased from Stressgen (Victoria, BC). Anti-protein disulfide-isomerase antibody was purchased from Millipore (Boston, MA). Anti-β-actin antibodies were purchased from Abmart (Xuhuidistrict, SH). Anti-mouse secondary antibody and anti-rabbit secondary antibody were purchased from Cell Signaling Technology (Beverly, MA). Pronase E was purchased from Roche (South San Francisco, CA). The cell lysis buffer for Western, IP and the BCA protein assay kit were purchased from Biyuntian (Haimen, Jiangsu, China). Hank's Buffered Salt Solution (HBSS) was purchased from Gibco (Grand Island, NY). Protease inhibitor cocktail and the protein A/G agarose beads were purchased from Pierce (Rockford, IL). The SV Total RNA Isolation System and GoScript™ Reverse Transcription System were purchased from Promega (Madison, WI).

### Cell Culture and Sample Preparation

THP-1 and other cell lines were obtained from the cell bank of the Chinese Academy of Sciences (Shanghai, China). Cells were grown in RPMI-1640 medium supplemented with 10% fetal bovine serum and 1% penicillin/streptomycin at 37°C in a humidified incubator with 5% CO_2_. Cells were treated with 5-azacytidine, H_2_O_2_, or gossypol dissolved in dimethyl sulfoxide and control cells were treated with the same amount of DMSO for the same time periods. After treatments, cells were washed twice with ice-cold PBS and lysed with RIPA lysis buffer (25 mmol/L Tris-HCl pH 7.6, 150 mmol/L NaCl, 0.1% SDS, 1% NP-40, 1% sodium deoxycholate, 1 mmol/L PMSF, and Roche Complete Protease Inhibitor Cocktail) for 30 min on ice. Cell lysates were clarified by centrifugation at 14, 000×g for 20 min at 4°C. The protein concentration in the supernatant of each sample was determined using a BCA protein assay kit.

### Flow Cytometry Analysis of Azacytidine-induced Cell Death

Cells were treated with azacytidine at different concentrations and for different periods of time. After treatments, cells were harvested, washed with cold PBS and re-suspended in 1× annexin V binding buffer supplied with the Apoptosis & Necrosis Quantification kit. 5 µl of FITC-Annexin V and 5 µl of Ethidium Homodimer III solutions were then added to each tube. After incubation at room temperature for 15 min in the dark, 400 µl 1× annexin V binding buffer was added to each tube and the cells were analyzed with a BD FACSCalibur™ Flow Cytometer (FACSCalibur, BD Biosciences, San Jose, CA) within 1 h of staining.

### Protein Separation by 1D SDS-PAGE and Proteomics Analysis

Equal amount of proteins from untreated- and treated-samples (about 30 µg) were separated by 1D SDS-PAGE, respectively. The gel bands of interest were excised from the gel, reduced with 25 mM of DTT and alkylated with 55 mM iodoacetamide. In gel digestion was then carried out with sequencing grade modified trypsin in 50 mM ammonium bicarbonate at 37°C overnight. The peptides were extracted twice with 0.1% trifluoroacetic acid in 50% acetonitrile aqueous solution for 30 min. Extracts were then centrifuged in a speedvac to reduce the volume.

For LC-MS/MS analysis, the digestion product was separated by a 65 min gradient elution at a flow rate 0.250 µl/min with an EASY-nLCII™ integrated nano-HPLC system (Proxeon, Denmark) which was directly interfaced with a Thermo LTQ-Orbitrap mass spectrometer. The analytical column was a home-made fused silica capillary column (75 µm ID, 150 mm length; Upchurch, Oak Harbor, WA) packed with C-18 resin (300 Å, 5 µm, Varian, Lexington, MA). Mobile phase A consisted of 0.1% formic acid, and mobile phase B consisted of 100% acetonitrile and 0.1% formic acid. The LTQ-Orbitrap mass spectrometer was operated in the data-dependent acquisition mode using Xcalibur 2.0.7 software and there was a single full-scan mass spectrum in the orbitrap (400–1800 m/z, 30,000 resolution) followed by 20 data-dependent MS/MS scans in the ion trap at 35% normalized collision energy (CID). The MS/MS spectra from each LC-MS/MS run were searched against the selected database using an in-house Proteome Discoverer searching algorithm.

The search criteria were as follows: full tryptic specificity was required; one missed cleavage was allowed; carbamidomethylation was set as the fixed modification; the oxidation (M) was set as the variable modification; precursor ion mass tolerances were set at 10 ppm for all MS acquired in an orbitrap mass analyzer; and the fragment ion mass tolerance was set at 0.8 Da for all MS^2^ spectra acquired in the linear ion trap.

### DNA Fragment Assay

DNA fragment assay was performed following the procedure described by Mazars et al [24]. Briefly, cells were washed with PBS twice and collected by centrifugation. Cells were suspended in 250 µl lysis buffer (1% NP-40, 20 mM EDTA, 50 mM Tris-HCl pH 7.5). The supernatants were collected by centrifugation for 5 min at 1,600×g. The supernatant was incubated with 0.71 mg/ml RNase A for 2 h at 56°C. Then 100 µg/ml pronase E was added and incubated with the supernatants overnight at 37°C. DNA fragments were precipitated with 0.5 volumes of 10 M ammonium acetate and 2 volumes of ethanol at −20°C for 12 h and centrifugation for 15 min at 15,000×g. The precipitate was washed with 70% ethanol and resuspended in loading buffer. Electrophoresis was performed in 0.5× Tris-borate-EDTA buffer for 30 min.

### Western Blot Analysis

Untreated- and azacytidine treated-THP-1 cells were collected and lysed on ice with Biyuntian cell lysis buffer for Western and IP supplied with the protease inhibitor cocktail. The supernatants were collected after centrifugation at 14,000×g for 10 min at 4°C. Protein concentrations were determined using the BCA protein assay kit. Proteins were separated on a 12% SDS-PAGE gel and transferred onto a PVDF transfer membrane by electroblotting. After blocking with 5% nonfat milk for 2 h at room temperature, the membrane was incubated overnight at 4°C with 1000× diluted primary antibody, washed with PBST buffer for 3 times, then incubated with 1000× diluted anti-mouse or anti-rabbit secondary antibody labeled with HRP at room temperature for 2 h. The membrane was further washed with PBST buffer 3 times and developed using ECL reagents (Engreen, China). β-actin was detected with anti-β-actin antibody as an internal control.

### Quantitative Real-time PCR (qPCR)

Cells were harvested after being treated with azacytidine for different periods of time. Total RNA was extracted by the SV Total RNA Isolation System. cDNA was synthesized from 0.8 µg total RNA using the GoScript™ Reverse Transcription System. All qPCR was performed using the Roche LightCycler® 480II Detection System with SYBR green incorporation according to the manufacturer's instructions. The primers were either designed by using the Primer Premier 5 software or from Primer Bank (http://pga.mgh.harvard.edu/primerbank/). To prevent amplification of genomic DNA, all target primers span exon-exon junctions. The specific PCR products were confirmed by melting curve analysis. Relative expression was analyzed using the 2^−ΔΔCt^ method. Primer sequences for qPCR are listed in Table S1.

### Drug Affinity Responsive Target Stability Assay (DARTS)

DARTS was carried out according to the published protocol [25]. Briefly, THP-1 cells were treated with 100 μΜ azacytidine for 12 h. Cells were harvested, washed with PBS twice, then lysed with Triton X-100 lysis buffer supplemented with protease and phosphatase inhibitors. After centrifugation at 14,000 rpm for 15 min, proteins from lysates were diluted to the same final concentration with Triton X-100 lysis buffer and TNC buffer containing 50 mM Tris-HCl and 50 mM NaCl. 10 mM CaCl_2_ was added to ensure that there was sufficient Ca^2+^ for pronase E to be fully active. All steps were performed on ice or at 4°C to prevent premature protein degradation. Each sample was then quickly warmed to room temperature and proteolysed with pronase E for 30 min. Digestion was stopped by directly adding the SDS loading buffer into each sample and heating to 95°C for 5 min. Samples were then separated by 1D SDS-PAGE followed by in-gel digestion and LC-MS/MS analysis.

### Co-immunoprecipitation

Cells were lysed with Biyuntian cell lysis buffer for Western and IP supplied with the protease inhibitor cocktail. The protein concentrations of the cell lysates were determined using a BCA assay. The protein A/G agarose beads were washed three times with cell lysis buffer. 2 mg of the cell lysate was incubated with 6 µg anti-bovine albumin antibody and 20 µl protein A/G agarose beads overnight at 4°C. The beads were centrifuged at 1000×g for 1 min and washed three times with the cell lysis buffer, and then proteins bound to the Protein A/G agarose beads were eluted by boiling the beads for 5 min in 1× SDS loading buffer. Each eluent was separated by 1D SDS-PAGE followed by western blot analysis.

### Detection of Reactive Oxygen Species (ROS) in Untreated and Azacytidine-treated Cells

ROS in untreated and azacytidine-treated cells was detected using an Image-iT™ LIVE Green Reactive Oxygen Species Detection Kit (Molecular Probes, Inc. Eugene, OR) following the manufacturer's instructions. Briefly, the cells were collected by centrifugation and washed once with warm HBSS/Ca/Mg. Cells were re-suspended with 500 µl of the 25 µM carboxy-H_2_DCFDA working solution for 25 min at 37°C, followed by addition of the Hoechst 33342 reagent to the reaction mixture at a final concentration of 1.0 µM and incubation for 5 min. The final products were washed gently with 1 ml HBSS/Ca/Mg immediately followed by imaging with Zeiss 710 Confocal Microscopy.

## Results and Discussion

### Azacytidine Induced Monocyte Death through Apoptosis and Necrosis

**Figure 1:**
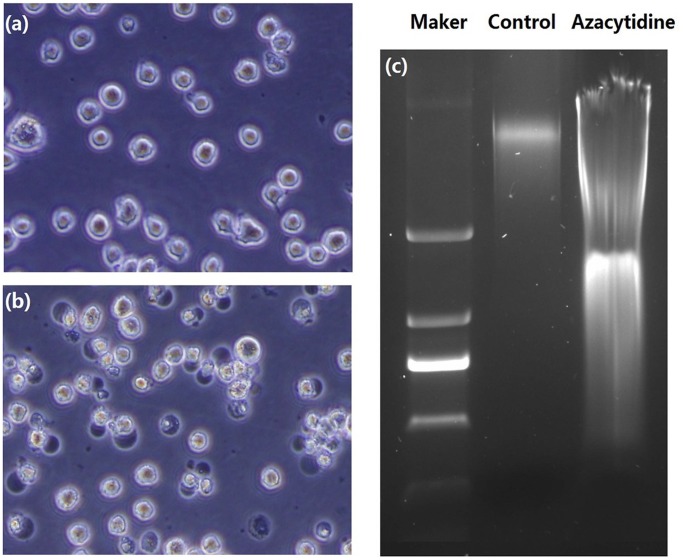
Morphologic images of untreated and azacytidine treated THP-1 cells. (a) Untreated cells; (b) cells being treated with 100 µM azacytidine for 24 h; and (c) Gel electrophoresis of DNA from untreated and 24 h 100 µM azacytidine-treated THP-1 cells.

A dose- and time-response study was performed and cell death was analyzed by FACS with FITC-Annexin V and Ethidium Homodimer III staining. Annexin V was used to stain apoptotic cells by binding to phosphatidylserine exposed on the outer leaflet, while Ethidium homodimer III was used to stain necrotic cells with red fluorescence. The quantitative FACS data representing the percentage of apoptotic and necrotic cells are shown in Figure S1. Apoptosis was the primary path to cell death when THP-1 cells were treated with 100 µM azacytidine for 4 h as shown in Figure S1(a). THP-1 cells exhibited a gradual increase in the necrotic fraction during this time. When THP-1 cells were treated for 12 h, the ratio of necrotic cells to apoptotic cells was approximately 2∶1, increasing to 3 to 1 after 24 hour treatment. These data demonstrated that necrosis became the major death pathway when THP-1 cells were treated with azacytidine for more than 12 h. Necrosis was also the primary process even when THP-1 cells were treated with lower concentrations of azacytidine for 24 h. See Figure S1(b). We estimated that the percentage of necrotic cells reached 60% after cells were treated with 100 µM azacytidine for 24 h. The morphological features of cell death were also consistent with necrosis in azacytidine-treated THP-1 and HL-60 cells. Images of cell morphology in untreated and 24 h azacytidine-treated cells are shown in Figure 1(a) and 1(b), respectively. The 24 h azacytidine-treated THP-1 cells displayed characteristic features of necrosis, including cell swelling and translucent cytoplasm with evidence of cell membrane disruption. The nuclei appeared pyknotic and there was considerable cellular debris in the pellets compared to the control sections. The DNA content of the necrotic cells was analyzed by gel electrophoresis. The gel image of DNA for untreated and azacytidine-treated cells (Figure 1(c)) shows that DNA from 24 h azacytidine-treated cells showed a random and general cleavage pattern and produced a smear that further confirmed that azacytidine-induced cell death occurs mainly via necrosis.

### Proteomic Analysis and Identification of Cell-bound Albumin in Necrotic Monocytes

**Figure 2:**
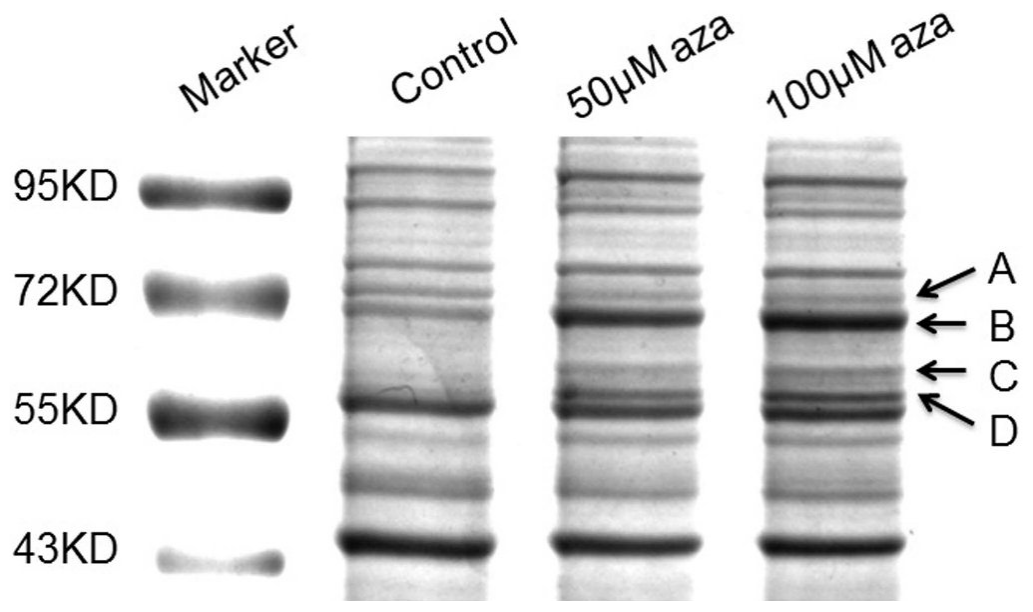
1D SDS-PAGE gel image of untreated and azacytidine-treated THP-1 cells. Lane 1, molecular weight markers; Lane 2, proteins from untreated cells; Lane 3, proteins from 24 h 50 µM azacytidine-treated cells; and Lane 4, proteins from 24 h 100 µM azacytidine-treated cells. Bands with differentially expressed proteins are labeled A, B, C, and D.

**Figure 3:**
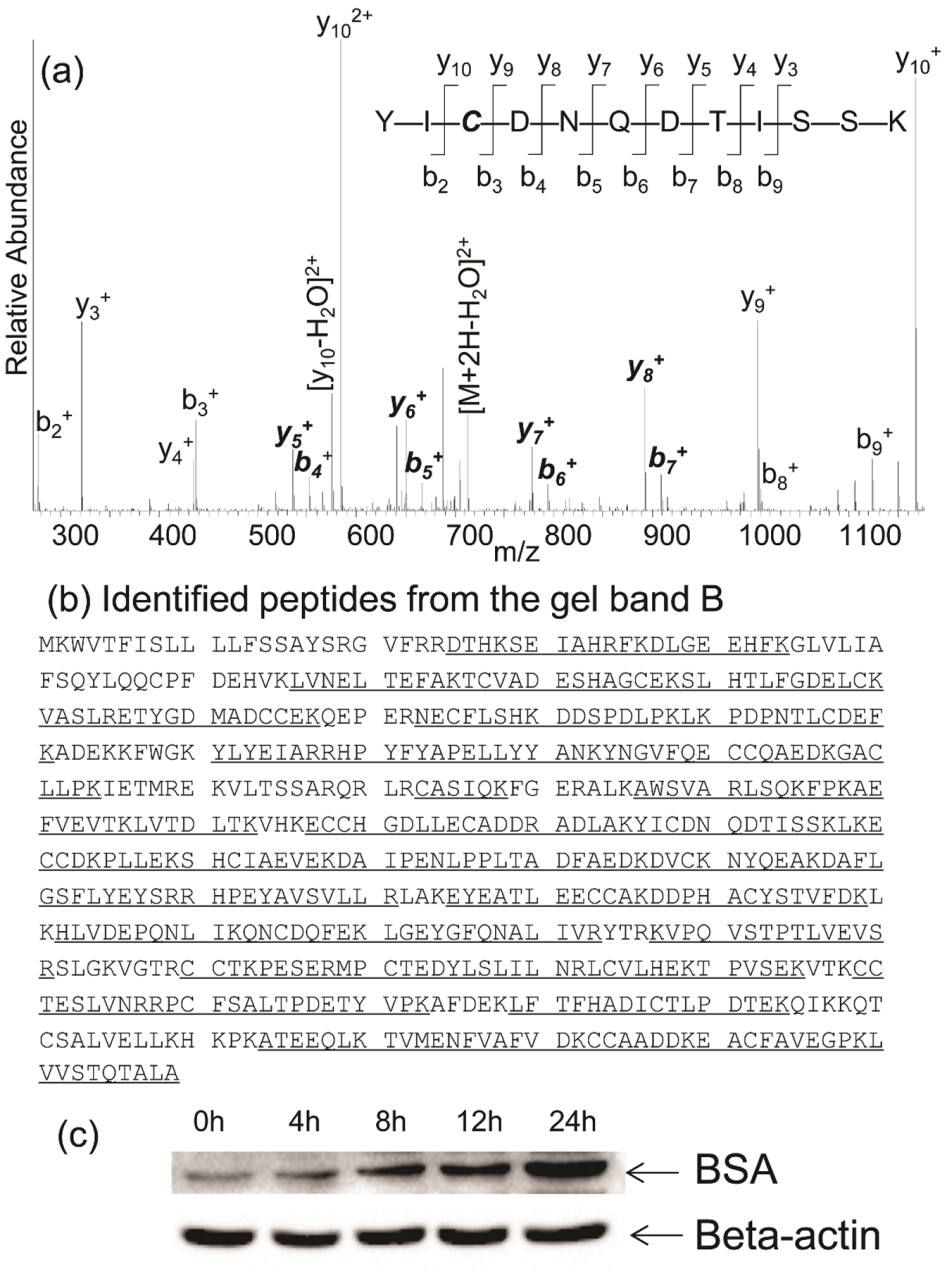
Identification of cell-bound BSA. (a) The MS/MS spectrum of a doubly charged ion at m/z 722.32 for MH_2_^2+^ corresponding to the mass of the peptide YICDNQDTISSK from bovine serum albumin. By searching a human protein database, this MS/MS spectrum matched YICENQDSISSK, which has an identical mass. The highlighted peaks matched only to the sequence of BSA. (b) Underlined peptides that were identified by MS/MS searching from the band B digestion cover 79% of the BSA sequence. (c) Western blot analysis of BSA from cells treated with 100 µM azacytidine for different periods of time. Lane 1, before treatment; Lane 2, 4 h; Lane 3, 8 h; Lane 4, 12 h; and Lane 5, 24 h.

Next, proteomic analysis was carried out on the necrotic monocytes. An equal amount of proteins from untreated- and 24 h azacytidine-treated THP-1 cells were loaded and separated by 1D SDS-PAGE (Figure 2). Differentially expressed proteins were immediately visible in four gel bands (labeled A, B, C, and D). The intensity of Band A was lighter after azacytidine treatment, indicating that azacytidine treatment down-regulates proteins in this band. The intensities of Bands B, C, and D were heavier after azacytidine treatment, indicating up-regulation of proteins in these bands. The intensity of Band B at about 70 kDa showed the largest change between untreated- and azacytidine-treated samples and became more intense with higher concentrations of azacytidine. Proteomic analysis and searching of a human protein database identified the major protein in this band as human albumin, production of which is a hallmark of hepatocytes. Previous studies have shown that monocytes are capable of differentiation into hepatocytes under different conditions and also that microglial cells in brain synthesized albumin [26]–[29]. In order to determine whether the cell-bound albumin originated from monocyte-derived hepatocytes, a human albumin-specific antibody was used to probe the identity of the cell-bound albumin. However, the antibody recognized the proteins in Band B as well as bovine serum albumin (data not shown), prompting us to verify that the MS/MS spectra matched that of human albumin. A representative MS/MS spectrum, which database searching matched to the tryptic peptide YICENQDSISSK of human albumin is shown in Figure 3(a). We noticed that the intense peaks in the MS/MS spectrum did not match to the expected b and y ions generated from fragmentation of the tryptic peptide of the human albumin, but rather that they perfectly matched to the similar peptide YICDNQDTISSK from bovine serum albumin (BSA), as highlighted in bold and italics. Indeed, searching a bovine protein database led to identification of BSA with sequence coverage of 79% of BSA sequence (Figure 3(b)), demonstrating that BSA is enriched in necrotic monocytes and originates from the cell culture medium. Using western blot analysis, we found the enrichment of cell-bound albumin was a time-dependent event (Figure 3(c)). Indeed, the intense band was no longer visible by 1D SDS-PAGE when THP-1 cells were cultured in serum-free medium and treated with 100 µM azacytidine.

### Monocyte–specific Enrichment of Cell-bound Albumin

Azacytidine also induced necrosis in other cell lines including A549, A2780 and HOC1a, and HUH-7 that are from lung cancer, ovarian cancer, and liver cancer, respectively. However, enrichment of the cell-bound albumin was not observed in these necrotic cells. 1D SDS-PAGE gel images of separated proteins from the necrotic A549 and A2780 cells are shown in Figure S2 and show no obvious differences in the intensity of the 70 kDa band, suggesting that the enrichment of the cell-bound albumin is unique to necrosis in monocytes.

We also noticed that Band B was more intense by 1D SDS-PAGE, while most other proteins were degraded after cells were treated with 100 µM azacytidine for 48 h, suggesting that the stability of the albumin might be increased by binding to a specific molecule. We next used DARTS to determine the binding partner of albumin [25]; this assay is based on a reduction in the protease susceptibility of the target protein upon binding to a specific molecule. Protein samples from untreated- and azacytidine-treated cells were incubated with pronase E for 30 min followed by protein separation on 1D SDS-PAGE (Figure S3(a)). Two intense bands were observed in the azacytidine-treated sample. Using proteomic analysis, we identified that the major protein in the upper band was BSA and that in the lower band was HSP60, suggesting that HSP60 may be a binding partner for BSA. To confirm the binding of BSA to HSP60, we used anti-BSA antibodies to immuno-precipitate BSA and associated proteins from untreated and azacytidine-treated THP-1 cells. Immunoprecipitated proteins were separated and probed with anti-HSP60 antibodies, and western blot analysis showed that HSP60 was immunoprecipitated by an anti-BSA antibody (Figure S3(b)). Previous studies reported that the cell-bound albumin in lymphocytes and macrophages was a peptidoglycan-, lipopolysacchride-, and lipoteichoic acid binding protein from cell culture medium [30]. Our data indicate that HSP60 is an additional binding partner of the cell-bound albumin, and that this complex retards degradation of albumin and HSP60.

### Overexpression and Ubiquitination of HSP60 and other ER-specific Chaperones

**Figure 4:**
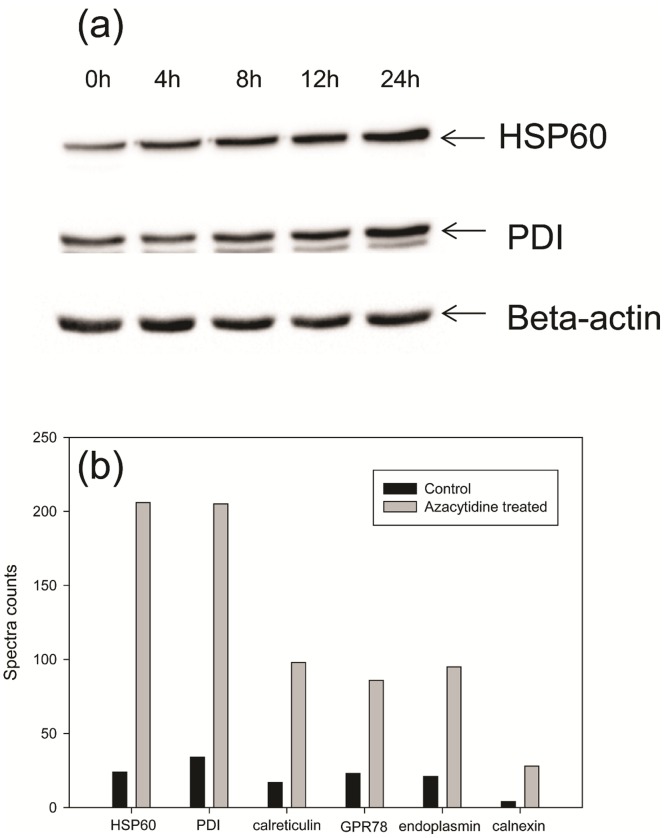
Changes of ER- and mitochondrial-chaperones upon azacytidine treatments. (a) Western blot analysis of HSP60 and PDI from cells treated with 100 µM azacytidine for different periods of time. Lane 1, before treatment; Lane 2, 4 h; Lane 3, 8 h; Lane 4, 12 h; and Lane 5, 24 h. (b) Relative abundance and fold of changes of ER- and mitochondrial chaperones from the untreated and azacytidine-treated monocytes. The number on the y axis represents spectra counts for each identified protein.

As shown in Figure 2, the intensities of two other bands below 70 kDa marked C and D, were also increased upon azacytidine-treatment. The major proteins in Band C and Band D were identified as HSP60 and protein disulfide isomerase (PDI), respectively. Using label free quantitation, we determined that the expression of HSP60 was increased 7-fold in the necrotic cells. HSP60 is a mitochondria-specific protein with multiple functions, and is essential in the synthesis and transportation of mitochondrial proteins from the cell's cytoplasm into the mitochondrial matrix [31]. HSP60 has been linked to diabetes, stress response, cancer and certain types of immunological disorders, and up-regulation of HSP60 is thought to sustain cellular survival under toxic or stressful circumstances [32]–[34]. We speculate that up-regulation of HSP60 may serve as the first line of defense against cytotoxicity elicited by azacytidine-induced cell stress. The up-regulated protein in Band D is an ER-specific chaperone, PDI, which catalyzes the formation and breakage of disulfide bonds to enable proteins to achieve their fully folded state, and plays a role in redox-mediated signaling transduction [35]–[36]. The up-regulation of HSP60 and PDI by azacytidine shows that the endoplasmic reticulum and mitochondria are the major targets of azacytidine-induced cell stress, and that both proteins may serve to protect the integrity of the endoplasmic reticulum and mitochondria under stress conditions. To confirm azacytidine-induced up-regulation of HSP60 and PDI, western blot analysis was carried out to study the expression changes of HSP60 and PDI. As shown in Figure 4(a), expression of HSP60 and PDI increased with time in 100 µM azacytidine-treated THP-1 cells.

We next identified all other differentially expressed proteins in whole gel lanes. The expressions of 15 proteins are significantly up-regulated upon azacytidine treatment (Table 1). Twelve of these are known ER- and mitochondrial-specific proteins, further indicating that these two organelles are major targets in oxidative stress-induced monocyte necrosis. It is worth mentioning that four other ER-specific chaperones, namely endoplasmin, calreticulin, glucose regulated protein 78 kDa (GRP 78) and calnexin, are all up-regulated upon azacytidine treatment. Endoplasmin is an abundant molecular chaperone that functions in the processing and transport of secreted proteins in ER and possesses ATPase activity and calcium-binding properties. Calreticulin is a lectin-like, calcium binding ER- specific chaperone that binds to misfolded proteins and prevents them from being exported from the ER to the Golgi apparatus. Overexpression of calreticulin in many cancer cells promotes macrophages to engulf hazardous cancerous cells [37]. GRP78 is a member of heat shock protein 70 family and acts as a central regulator of ER functions by participating in the ER protein folding and assembly process, and maintaining ER Ca^2+^ homeostasis, unfolded protein response and specific anti-apoptotic actions. Calnexin is also an endoplasmic reticulum specific chaperone, whose main function is to assist in protein folding and quality control. Calreticulin, calnexin and ERp57 constitute the calreticulin/calnexin cycle and function in the quality control of transmembrane and secreted glycoproteins in the ER. Analysis by label-free quantitation showed that HSP60 and PDI are equally abundant with other chaperones in untreated monocytes, but are twice as abundant as the other four chaperones after treatment with azacytidine (Figure 4(b)). Calnexin is the least abundant of the chaperones. All these chaperones assist in the protein folding and their over-expression indicates that ER proteins are the major targets of azacytidine-induced cellular stress.

**Table 1:** Up-regulated proteins after treatment with 100 µM azacytidine.

Protein ID	Description
IPI00784154	60 kDa heat shock protein, mitochondrial
IPI00010796	Protein disulfide-isomerase
IPI00303476	ATP synthase subunit beta, mitochondrial
IPI00291006	Malate dehydrogenase, mitochondrial
IPI00020599	Calreticulin
IPI00027230	Endoplasmin
IPI00003362	78 kDa glucose-regulated protein
IPI00011416	Delta(3,5)-Delta(2,4)-dienoyl-CoA isomerase, mitochondrial
IPI00440493	ATP synthase subunit alpha, mitochondrial
IPI00939159	Adenylyl cyclase-associated protein
IPI01014382	highly similar to Glutamate dehydrogenase 1, mitochondrial
IPI00941747	Calnexin
IPI00024911	Endoplasmic reticulum resident protein 29
IPI00383581	highly similar to Neutral alpha-glucosidase AB
IPI00027180	CAAX prenyl protease 1 homolog

**Figure 5:**
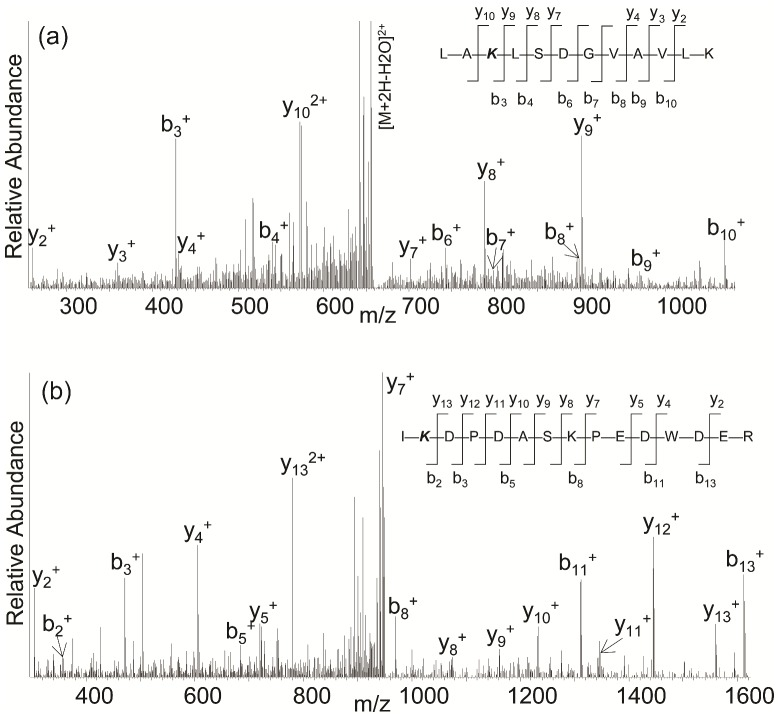
MS/MS spectra of ubiquitinated peptides from HSP60 and calreticulin. (a) The MS/MS spectrum of a doubly charged ion at m/z 664.40 for MH_2_^2+^ corresponding to the mass of the ubiquitinated peptide LAKLSDGVAVLK. The labeled peaks correspond to masses of y and b ions of the modified peptide; (b) The MS/MS spectrum of a doubly charged ion at m/z 957.95 for MH_2_^2+^ corresponding to the mass of the ubiquitinated peptide IKDPDASKPEDWDER. The labeled peaks correspond to masses of y and b ions of the modified peptide.

Furthermore, we also found that azacytidine induced HSP60 ubiquitination at Lys-396 residue (Figure 5(a)). The major fragment ions matched to the expected b and y ions of LA**K(GG)**LSDGVAVLK, and ubiquitination of HSP60 was not found in the untreated sample. It is not clear what role ubiquitination plays in stress-induced monocyte necrosis. Calreticulin was also ubiquitinated at Lys-209 residue only in necrotic monocytes (Figure 5(b)) and the major fragment ions matched to the expected b and y ions of I**K(GG)**DPDASKPEDWDER. The ubiquitination of calreticulin may play a role in the stress-activated ubiquitin–proteasome pathway.

### HSP71 and HSP90 being Released from Necrotic Monocytes into the Cell Medium

**Figure 6:**
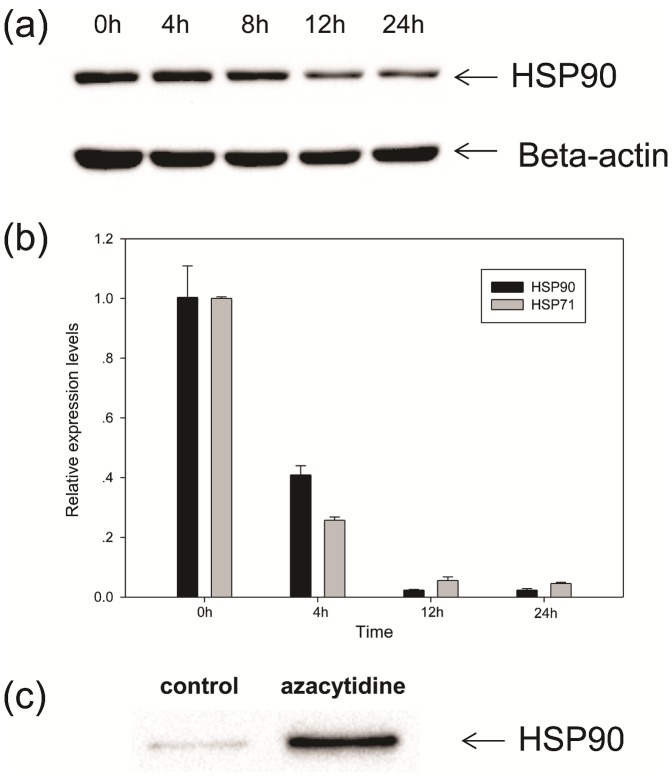
Changes of HSP71 and HSP90 in cells and culture medium upon azacytidine treatments. (a) Western blot analysis of HSP90 from cells treated with 100 µM azacytidine for different periods of time. Lane 1, before treatment; Lane 2, 4 h; Lane 3, 8 h; Lane 4, 12 h; and Lane 5, 24 h. (b) qPCR analysis of HSP90 and HSP71 from cells treated with 100 µM azacytidine for different periods of time. (c) Western blot analysis of HSP90 from cell culture medium. Lane 1, medium of untreated cells; Lane 2, medium from cells treated with 100 µM azacytidine for 24 h.

As shown in Figure 2, the intensity of Band A was decreased upon azacytidine-treatment. The major proteins in this band were identified as HSP71 and HSP90. The decrease of HSP90 expression was confirmed by western blot analysis (Figure 6(a)). A significant decrease in HSP90 was observed when THP-1 cells were treated with 100 µM azacytidine for 12 h concomitant with the shift of apoptosis to necrosis for azacytidine-treated cells. HSP90 is one of the most abundant cytosolic chaperones in cells with multiple functions in assisting protein folding, stabilizing various proteins, and aiding protein degradation. To confirm that azacytidine induced down-regulation of HSP90, qPCR analysis was carried out, and showed that the expression of HSP90 at the mRNA level is down-regulated after azacytidine treatment (Figure 6(b)). HSP71 is the major chaperone involving protein folding and is a member of the heat shock protein 70 family. In cooperation with other chaperones, HSP71 stabilizes proteins against aggregation and mediates the folding of newly-translated polypeptides in the cytosol through its ability to recognize non-native protein conformations. The expression of HSP71 at the mRNA level was also down-regulated after azacytidine treatment, as determined by qPCR analysis (Figure 6(b)).

Several studies detected HSP71 outside cells, where it is thought to activate the immune system [38]–[40] it has also been suggested that necrotic cells release HSP71 into the extracellular medium [38]. We carried out a proteomic analysis of the culture medium of necrotic- and untreated cells, and found that the concentrations of HSP71 and HSP90 were much higher in the medium of necrotic cells than those of the untreated cells. This was confirmed by western blot analysis of the culture medium of untreated and azacytidine-treated THP-1 cells (Figure 6(c)). Our results provide direct evidence that necrotic monocytes release HSP71 and HSP90 into the cell culture medium. HSP71 does not have a consensus secretary signal, and the mechanism for translocation of this protein across membranes may involve in binding of HSP71 with the plasma membrane before release into the extracellular environment. The cell membrane rupture in necrotic monocytes (Figure 1(b)) may be involved in the release of HSP71 and HSP90 into the cell culture medium. Therefore, down-regulation of HSP90 and HSP71 at the mRNA level, and release of HSP90 and HSP71 into the cell medium, together result in the azacytidine-induced decrease of HSP90 and HSP71 proteins.

Azacytidine also induced down-regulation of 30 other proteins as listed in Table 2. Eleven of these are cytosolic proteins, including ADP-ribosylation factor 1, Isoform 1 of Triosephosphate isomerase, Isoform alpha-enolase of Alpha-enolase, Elongation factor 1-alpha 1, HSP71, HSP90 alpha, Plastin-2, Isoform 1 of Myosin-9, Isoform M2 of pyruvate kinase isozymes M1/M2, Peptidyl-prolylcis-trans isomerase A (cyclophilin A), and beta tubulin. Cyclophilin A is a peptidyl-prolyl isomerase with chaperone functions. Four proteins are associated with chromatin, namely H2A, H2B, H4 and Nucleophosmin. Nucleophosmin is a histone chaperone located in the nucleolus, but translocates to the nucleoplasm during serum starvation or treatment with anticancer drugs. It has multiple functions such as ribosome biogenesis and centrosome duplication, and modulates the activity of certain tumor suppressor genes, such as p53 and Arf. The present study therefore shows that cytosolic and nuclear chaperones are down-regulated upon azacytidine treatment, while ER- and mitochondrial chaperones are up-regulated. The other down-regulated proteins participate in diverse cellular processes such as glycolysis, protein folding, and cytoskeletal organization.

**Table 2:** Down-regulated proteins after treatment with 100 µM azacytidine.

Protein ID	Description
IPI00396485	Elongation factor 1-alpha 1
IPI00025416	Actin, gamma-enteric smooth muscle
IPI00465248	Isoform alpha-enolase of Alpha-enolase
IPI00414676	Heat shock protein HSP 90-beta
IPI00018534	Histone H2B type 1-L
IPI00645452	Tubulin, beta
IPI00792677	Highly similar to Tubulin alpha-ubiquitous chain
IPI00479186	Isoform M2 of Pyruvate kinase isozymes M1/M2
IPI00795257	Glyceraldehyde-3-phosphate dehydrogenase
IPI00219757	Glutathione S-transferase P
IPI00453473	Histone H4
IPI00419585	Peptidyl-prolylcis-trans isomerase A
IPI00081836	Histone H2A type 1-H
IPI00216691	Profilin-1
IPI00019502	Isoform 1 of Myosin-9
IPI00784295	Isoform 1 of heat shock protein HSP 90-alpha
IPI00550363	Transgelin-2
IPI00003865	Isoform 1 of Heat shock cognate 71 kDa protein
IPI00465439	Fructose-bisphosphatealdolase A
IPI00219219	Galectin-1
IPI00000874	Peroxiredoxin-1
IPI00797270	Isoform 1 of Triosephosphate isomerase
IPI00010471	Plastin-2
IPI00797148	Isoform 2 of Heterogeneous nuclear ribonucleoprotein A1
IPI00169383	Phosphoglycerate kinase 1
IPI00220740	Isoform 2 of nucleophosmin
IPI00215914	ADP-ribosylation factor 1
IPI00218414	Carbonic anhydrase 2
IPI00795292	Isoform 3 of nucleoside diphosphate kinase B
IPI00646304	Peptidyl-prolylcis-trans isomerase B

### Azacytidine-induced Monocyte Necrosis through Oxidative Stress

**Figure 7:**
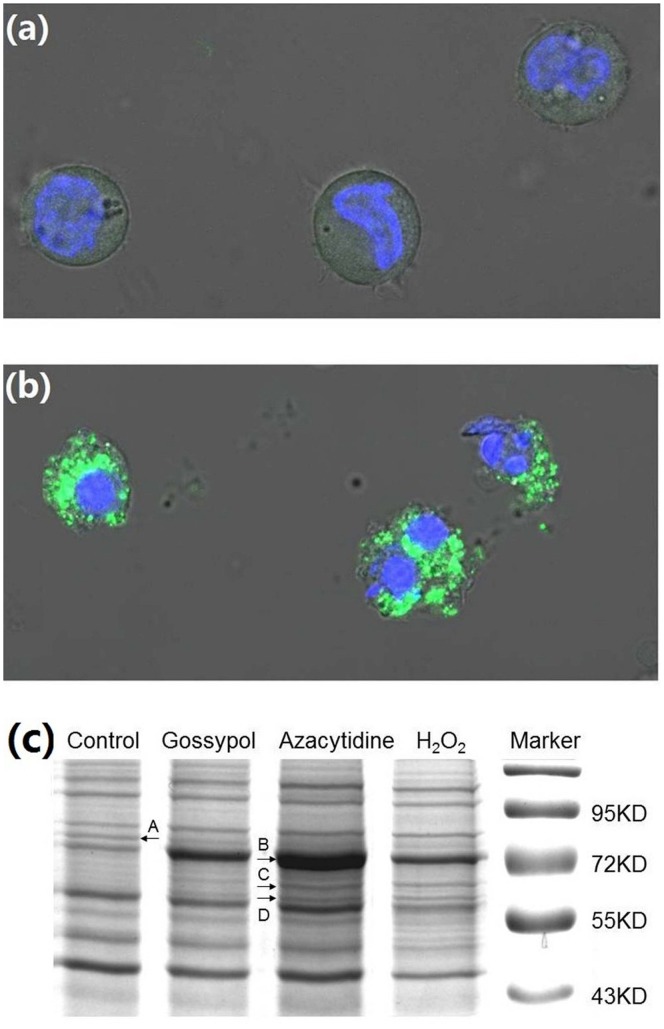
Detection of ROS in untreated and azacytidine-treated THP-1 cells. Cells were labeled with carboxy-H_2_DCFDA, which exhibits green fluorescence upon reaction with ROS, and nuclei were stained with blue-fluorescent Hoechst 33342. (a) Untreated THP-1 cells; and (b) azacytidine-treated THP-1 cells. (c) The 1D-SDS-PAGE gel image of proteins from untreated, gossypol-, azacytidine- or H_2_O_2_-treated THP-1 cells. Lane 1, proteins from untreated cells; Lane 2, proteins from 20 µM gossypol-treated cells; Lane 3, proteins from 100 µ M azacytidine-treated cells; Lane 4, proteins from 50 mM H_2_O_2_-treated cells. The bands with differentially expressed proteins are labeled A, B, C, and D.

Azacytidine is a DNA methyltransferase inhibitor and has been used for treatment of myelodysplastic syndromes and acute myeloid leukemias [41]–[43]. In addition to its roles in inhibition of DNA methyltransferase and reorganization of genomic histone modification patterns [44]–[48], the ROS-inducing activity of azacytidine has also been amply documented. Azacytidine as a single agent, or combined with other anti-tumor agents, generates ROS in tumor cell lines, and the production of ROS in acute myeloid leukemia and acute lymphoblastic leukemia cells has been shown to be an indicator for the synergistic cytotoxic effects of azacytidine [49]–[51]. Therefore, the necrosis of monocytes may be attributed to ROS generated by azacytidine treatment. In the present study, an Image-iT LIVE Reactive Oxygen Species Kit was used to detect ROS in the untreated and azacytidine treated cells. Cells were labeled with carboxy-H_2_DCFDA, which fluoresces when oxidized by ROS, and nuclei were stained with blue-fluorescent Hoechst 33342. The azacytidine-treated cells exhibited much stronger green fluorescence (Figure 7(b)) in comparison to untreated cells, indicating that azacytidine induced a significant increase in ROS.

To further support this hypothesis, THP-1 cells were also treated with hydrogen peroxide or gossypol. We found that hydrogen peroxide- or gossypol-treatment resulted in cell necrosis with identical morphological changes and a random cleavage pattern of DNA as seen in azacytidine-treated cells. Furthermore, treatment of THP-1 cells with hydrogen peroxide or gossypol resulted in similar protein band patterns on 1D SDS-PAGE as shown in Figure 7(c). Both hydrogen peroxide and gossypol induce enrichment of cell-bound albumin and up-regulation of ER- and mitochondria-specific heat shock proteins. Gossypol is a natural polyphenolic compound that is extracted from cotton and the tropical plants and which possesses anti-proliferative properties and has been employed as an anti-cancer agent [52]–[53]. Although gossypol induces apoptosis through inhibition of antiapoptotic Bcl-2 proteins and activation of caspase-3 [54], it also stimulates production of ROS [55]. The present study demonstrates that necrosis induced by azacytidine, hydrogen peroxide or gossypol has a common mechanism of action, namely oxidative stress. Although great efforts are still needed to understand the molecular mechanisms of necrosis, our data demonstrate that monocyte necrosis involves the following sequence of events: generation of ROS upon azacytidine, hydrogen peroxide or gossypol treatment; ROS attacking ER and mitochondrial proteins; activating redox signaling pathways and leading to up-regulation of ER and mitochondrial chaperones; and ROS-induced plasma membrane rupture, leading to the release of HSP90 and HSP71 into the cell culture medium. The consequences of oxidative stress in monocyte necrosis are illustrated in Figure S4.

### Conclusion

In the present study, we show that azacytidine induces monocyte necrosis through an oxidative stress mechanism, resulting in up-regulation of ER- and mitochondria-specific chaperones including HSP60, PDI, calreticulin, endoplasmin, GRP78 and calnexin, and enrichment of the cell-bound albumin. Azacytidine induces the highest expression changes and ubiquitination of HSP60, indicating that HSP60 plays an important role in monocyte necrosis. The expression levels of cytosolic chaperones HSP71 and HSP90 are lower after azacytidine treatment, concomitant with the increase of these chaperones in the cell culture medium, providing direct evidence that necrotic monocytes release HSP90 and HSP71 into the cell culture medium. Enrichment of the cell-bound albumin is a characteristic of monocyte-specific necrosis and HSP60 may represent a new target for leukemia treatment.

## Supporting Information

Figure S1 **FACS analysis of azacytidine-induced cell death.** THP-1 cells were treated with azacytidine at different concentrations for different periods of time. Apoptotic and necrotic cells were stained with FITC-Annexin V and Ethidium Homodimer III, respectively. (a) Percentages of apoptotic- and necrotic-cells from 100 µM azacytidine-treated cells for 0, 4, 12, and 24 h. (b) Percentages of apoptotic- and necrotic-cells from untreated, 20, 60, and 100 µM azacytidine-treated cells for 24 h. Results are expressed as the mean of three experiments.
(PDF)

Figure S2 **1D-SDS-PAGE gel image of protein samples from untreated and gossypol or azacytidine-treated A549 and A2780 cells.** Lane 1, Molecular markers; Lane 2, protein from untreated A549 cells; Lane 3 and 4, proteins from 20 µM gossypol- and 100 µM azacytidine-treated A549 cells; Lane 5, protein from untreated A2780 cells, Lane 6 and 7, proteins from 20 µM gossypol- and 100 µM azacytidine-treated A2780 cells.
(TIF)

Figure S3 **DARTS and IP analysis of HSP60 binding to BSA.** (a) 1D SDS-PAGE gel image of proteins from pronase E treated samples. Lane 1, molecular weight markers; Lane 2, proteins from untreated cells; and Lane 3, proteins from azacytidine-treated cells. The bands of interest are marked with arrows. (b) Western blot analysis of HSP60 after anti-BSA antibody-immunoprecipitation.
(TIF)

Figure S4 **Model of azacytidine-induced changes in necrotic monocytes.**
(TIF)

Table S1 **Primers used for qPCR analysis in this work.**
(DOCX)

## References

[1] Edinger AL, Thompson CB (2004) Death by design: apoptosis, necrosis and autophagy. Current opinion in cell biology 16: 663–669. 15530778 10.1016/j.ceb.2004.09.011

[2] Zong WX, Thompson CB (2006) Necrotic death as a cell fate. Genes & development 20: 1–15. 16391229 10.1101/gad.1376506

[3] Golstein P, Kroemer G (2007) Cell death by necrosis: towards a molecular definition. Trends in biochemical sciences 32: 37–43. 17141506 10.1016/j.tibs.2006.11.001

[4] Han J, Zhong CQ, Zhang DW (2011) Programmed necrosis: backup to and competitor with apoptosis in the immune system. Nature immunology 12: 1143–1149. 22089220 10.1038/ni.2159

[5] Wang Z, Jiang H, Chen S, Du F, Wang X (2012) The mitochondrial phosphatase PGAM5 functions at the convergence point of multiple necrotic death pathways. Cell 148: 228–243. 22265414 10.1016/j.cell.2011.11.030

[6] Rock KL, Kono H (2008) The inflammatory response to cell death. Annual review of pathology 3: 99–126. 10.1146/annurev.pathmechdis.3.121806.151456 PMC3094097 18039143

[7] Jäättelä M, Tschopp J (2003) Caspase-independent cell death in T lymphocytes. Nature immunology 4: 416–423. 12719731 10.1038/ni0503-416

[8] Lyer SS, Pulskens WP, Sadler JJ, Butter LM, Teske GJ, et al (2009) Necrotic cells trigger a sterile inflammatory response through the Nlrp3 inflammasome. Science's STKE 106: 20388–20393. 10.1073/pnas.0908698106 PMC2787135 19918053

[9] Festjens N, Vanden Berghe T, Vandenabeele P (2006) Necrosis, a well-orchestrated form of cell demise: signalling cascades, important mediators and concomitant immune response. Biochimica et Biophysica Acta (BBA)-Bioenergetics 1757: 1371–1387. 16950166 10.1016/j.bbabio.2006.06.014

[10] Basu S, Binder RJ, Suto R, Anderson KM, Srivastava PK (2000) Necrotic but not apoptotic cell death releases heat shock proteins, which deliver a partial maturation signal to dendritic cells and activate the NF-κB pathway. International immunology 12: 1539–1546. 11058573 10.1093/intimm/12.11.1539

[11] Basu S, Srivastava PK (2000) Heat shock proteins: the fountainhead of innate and adaptive immune responses. Cell stress & chaperones 5: 443–451. 11189450 10.1379/1466-1268(2000)005<0443:hsptfo>2.0.co;2 PMC312875

[12] Lipscomb MF, Masten BJ (2002) Dendritic cells: immune regulators in health and disease. Physiological reviews 82: 97–130. 11773610 10.1152/physrev.00023.2001

[13] Robert J (2003) Evolution of heat shock protein and immunity. Developmental & Comparative Immunology 27: 449–464. 12697304 10.1016/s0145-305x(02)00160-x

[14] Mark G, Zihai L (2009) Heat-shock proteins in infection-mediated inflammation-induced tumorigenesis.Journal of Hematology &. Oncology30: 5. 10.1186/1756-8722-2-5 PMC2644312 19183457

[15] Tsan MF, Gao B (2004) Heat shock protein and innate immunity. Cell Mol Immunol 1: 274–279. 16225770

[16] Beg AA (2002) Endogenous ligands of Toll-like receptors: implications for regulating inflammatory and immune responses. Trends in immunology 23: 509–512. 12401394 10.1016/s1471-4906(02)02317-7

[17] Calderwood SK, Murshid A, Gong J (2012) Heat shock proteins: conditional mediators of inflammation in tumor immunity. Frontiers in Immunology 3: 75. 22566956 10.3389/fimmu.2012.00075 PMC3342006

[18] Kappler M, Gerry AB, Brown E, Reid L, Leake DS, et al (2007) Aqueous peroxyl radical exposure to THP-1 cells causes glutathione loss followed by protein oxidation and cell death without increased caspase-3 activity. Biochimica et Biophysica Acta (BBA)-Molecular Cell Research 1773: 945–953. 17509699 10.1016/j.bbamcr.2007.04.001

[19] McComb S, Cheung H, Korneluk R, Wang S, Krishnan L, et al (2012) cIAP1 and cIAP2 limit macrophage necroptosis by inhibiting Rip1 and Rip3 activation. Cell Death & Differentiation 19: 1791–1801. 22576661 10.1038/cdd.2012.59 PMC3469059

[20] Wong KW, Jacobs Jr WR (2011) Critical role for NLRP3 in necrotic death triggered by Mycobacterium tuberculosis. Cellular microbiology 13: 1371–1384. 21740493 10.1111/j.1462-5822.2011.01625.x PMC3257557

[21] Fujisawa A, Kambe N, Saito M, Nishikomori R, Tanizaki H, et al (2007) Disease-associated mutations in CIAS1 induce cathepsin B-dependent rapid cell death of human THP-1 monocytic cells. Blood 109: 2903–2911. 17164343 10.1182/blood-2006-07-033597

[22] Morinaga Y, Yanagihara K, Nakamura S, Hasegawa H, Seki M, et al (2010) Legionella pneumophila induces cathepsin B-dependent necrotic cell death with releasing high mobility group box1 in macrophages. Respiratory Research 11: 158. 21092200 10.1186/1465-9921-11-158 PMC3003236

[23] Imre G, Dunai Z, Petak I, Mihalik R (2007) Cystein cathepsin and Hsp90 activities determine the balance between apoptotic and necrotic cell death pathways in caspase-compromised U937 cells. Biochimica et Biophysica Acta (BBA)-Molecular Cell Research 1773: 1546–1557. 17707089 10.1016/j.bbamcr.2007.07.003

[24] Mazars A, Lallemand F, Prunier C, Marais J, Ferrand N, et al (2001) Evidence for a role of the JNK cascade in Smad7-mediated apoptosis. Journal of Biological Chemistry 276: 36797–36803. 11477069 10.1074/jbc.M101672200

[25] Lomenick B, Hao R, Jonai N, Chin RM, Aghajan M, et al (2009) Target identification using drug affinity responsive target stability (DARTS). Proceedings of the National Academy of Sciences 106: 21984–21989. 10.1073/pnas.0910040106 PMC2789755 19995983

[26] Zhao Y, Glesne D, Huberman E (2003) A human peripheral blood monocyte-derived subset acts as pluripotent stem cells. Proceedings of the National Academy of Sciences 100: 2426–2431. 10.1073/pnas.0536882100 PMC151357 12606720

[27] Ruhnke M, Ungefroren H, Nussler A, Martin F, Brulport M, et al (2005) Differentiation of In Vitro–Modified Human Peripheral Blood Monocytes Into Hepatocyte–like and Pancreatic Islet-like Cells. Gastroenterology 128: 1774–1786. 15940611 10.1053/j.gastro.2005.03.029

[28] Yan L, Han Y, Wang J, Liu J, Hong L, et al (2007) Peripheral blood monocytes from patients with HBV related decompensated liver cirrhosis can differentiate into functional hepatocytes. American journal of hematology 82: 949–954. 17724706 10.1002/ajh.21030

[29] Ahn SM, Byun K, Cho K, Kim JY, Yoo JS, et al (2008) Human microglial cells synthesize albumin in brain. PLoS One 3: e2829. 18665237 10.1371/journal.pone.0002829 PMC2483733

[30] Dziarski R (1994) Cell-bound albumin is the 70-kDa peptidoglycan-, lipopolysaccharide-, and lipoteichoic acid-binding protein on lymphocytes and macrophages. Journal of Biological Chemistry 269: 20431–20436. 8051139

[31] Koll H, Guiard B, Rassow J, Ostermann J, Horwich A, et al (1992) Antifolding activity of hsp60 couples protein import into the mitochondrial matrix with export to the intermembrane space. Cell 68: 1163–1175. 1347713 10.1016/0092-8674(92)90086-r

[32] Calabrese V, Mancuso C, Ravagna A, Perluigi M, Cini C, et al (2007) In vivo induction of heat shock proteins in the substantia nigra following L-DOPA administration is associated with increased activity of mitochondrial complex I and nitrosative stress in rats: regulation by glutathione redox state. Journal of neurochemistry 101: 709–717. 17241115 10.1111/j.1471-4159.2006.04367.x

[33] Rossi MR, Somji S, Garrett SH, Sens MA, Nath J, et al (2002) Expression of hsp 27, hsp 60, hsc 70, and hsp 70 stress response genes in cultured human urothelial cells (UROtsa) exposed to lethal and sublethal concentrations of sodium arsenite. Environmental health perspectives 110: 1225–1232. 12460802 10.1289/ehp.021101225 PMC1241110

[34] Chandra D, Choy G, Tang DG (2007) Cytosolic Accumulation of HSP60 during Apoptosis with or without Apparent Mitochondrial Release: evidence that its pro-apoptotic or pro-survival functions involve differential interactions with caspase-3. Journal of Biological Chemistry282: 31289–31301. 10.1074/jbc.M702777200 17823127

[35] Wilkinson B, Gilbert HF (2004) Protein disulfide isomerase. Biochimica et Biophysica Acta (BBA)-Proteins & Proteomics 1699: 35–44. 15158710 10.1016/j.bbapap.2004.02.017

[36] Gruber CW, Cemazar M, Heras B, Martin JL, Craik DJ (2006) Protein disulfide isomerase: the structure of oxidative folding. Trends in biochemical sciences 31: 455–464. 16815710 10.1016/j.tibs.2006.06.001

[37] Gold LI, Eggleton P, Sweetwyne MT, Van Duyn LB, Greives MR, et al (2010) Calreticulin: non-endoplasmic reticulum functions in physiology and disease. The FASEB Journal 24: 665–683. 19940256 10.1096/fj.09-145482 PMC2830142

[38] Vega VL, Rodríguez-Silva M, Frey T, Gehrmann M, Diaz JC, et al (2008) Hsp70 translocates into the plasma membrane after stress and is released into the extracellular environment in a membrane-associated form that activates macrophages. The Journal of Immunology 180: 4299–4307. 18322243 10.4049/jimmunol.180.6.4299

[39] Asea A (2007) Release of Heat Shock Proteins: Passive Versus Active Release Mechanisms. Heat shock proteins 1: 3–20.

[40] Asea A (2007) Mechanisms of HSP72 release. Journal of biosciences 32: 579–584. 17536177 10.1007/s12038-007-0057-5

[41] Fenaux P, Mufti GJ, Hellstrom-Lindberg E, Santini V, Finelli C, et al (2009) Efficacy of azacitidine compared with that of conventional care regimens in the treatment of higher-risk myelodysplastic syndromes: a randomised, open-label, phase III study. The lancet oncology 10: 223–232. 19230772 10.1016/S1470-2045(09)70003-8 PMC4086808

[42] Silverman LR, McKenzie DR, Peterson BL, Holland JF, Backstrom JT, et al (2006) Further analysis of trials with azacitidine in patients with myelodysplastic syndrome: studies 8421, 8921, and 9221 by the Cancer and Leukemia Group B. Journal of Clinical oncology. 24: 3895–3903. 10.1200/JCO.2005.05.4346 16921040

[43] Silverman LR, Demakos EP, Peterson BL, Kornblith AB, Holland JC, et al (2002) Randomized controlled trial of azacitidine in patients with the myelodysplastic syndrome: a study of the cancer and leukemia group B. Journal of Clinical oncology. 20: 2429–2440. 10.1200/JCO.2002.04.117 12011120

[44] Follo MY, Finelli C, Mongiorgi S, Clissa C, Bosi C, et al (2009) Reduction of phosphoinositide-phospholipase C beta1 methylation predicts the responsiveness to azacitidine in high-risk MDS. Proceedings of the National Academy of Sciences 106: 16811–16816. 10.1073/pnas.0907109106 PMC2741479 19805378

[45] Hagemann S, Heil O, Lyko F, Brueckner B (2011) Azacytidine and decitabine induce gene-specific and non-random DNA demethylation in human cancer cell lines. PLoS One 6: e17388. 21408221 10.1371/journal.pone.0017388 PMC3049766

[46] Christman JK (2002) 5-Azacytidine and 5-aza-2′-deoxycytidine as inhibitors of DNA methylation: mechanistic studies and their implications for cancer therapy. Oncogene 21: 5483–5495. 12154409 10.1038/sj.onc.1205699

[47] Tran HTT, Kim HN, Lee IK, Kim YK, Ahn JS, et al (2011) DNA methylation changes following 5-azacitidine treatment in patients with myelodysplastic syndrome. Journal of Korean medical science 26: 207–213. 21286011 10.3346/jkms.2011.26.2.207 PMC3031004

[48] Komashko VM, Farnham PJ (2010) 5-azacytidine treatment reorganizes genomic histone modification patterns. Epigenetics: official journal of the DNA Methylation Society 5: 229–240. 10.4161/epi.5.3.11409 20305384

[49] Chandra J (2009) Oxidative stress by targeted agents promotes cytotoxicity in hematologic malignancies. Antioxidants & redox signaling 11: 1123–1137. 19018667 10.1089/ars.2008.2302 PMC2842131

[50] Gao S, Mobley A, Miller C, Boklan J, Chandra J (2008) Potentiation of reactive oxygen species is a marker for synergistic cytotoxicity of MS-275 and 5-azacytidine in leukemic cells. Leukemia research 32: 771–780. 18031811 10.1016/j.leukres.2007.09.007 PMC2320596

[51] Nadasi E, Clark JS, Szanyi I, Varjas T, Ember I, et al (2009) Epigenetic Modifiers Exacerbate Oxidative Stress in Renal Proximal Tubule Cells. Anticancer research 29: 2295–2299. 19528494

[52] Stein RC, Joseph A, Matlin SA, Cunningham DC, Ford HT, et al (1992) A preliminary clinical study of gossypol in advanced human cancer. Cancer chemotherapy and pharmacology 30: 480–482. 1394805 10.1007/BF00685601

[53] Varol U, Karaca B, Tunali D, Degirmenci M, Cirak Y, et al (2009) The effect of racemic gossypol and at-101 on angiogenic profile of ovcar-3 cells: a preliminary molecular framework for gossypol enantiomers. Experimental oncology 31: 220–225. 20010531

[54] Meng Y, Tang W, Dai Y, Wu X, Liu M, et al (2008) Natural BH3 mimetic (-)-gossypol chemosensitizes human prostate cancer via Bcl-xL inhibition accompanied by increase of Puma and Noxa. Molecular cancer therapeutics 7: 2192–2202. 18645028 10.1158/1535-7163.MCT-08-0333 PMC2515935

[55] Sung B, Ravindran J, Prasad S, Pandey MK, Aggarwal BB (2010) Gossypol induces death receptor-5 through activation of the ROS-ERK-CHOP pathway and sensitizes colon cancer cells to TRAIL. Journal of Biological Chemistry 285: 35418–35427. 20837473 10.1074/jbc.M110.172767 PMC2975165

