# Supplementary figures and images for: Oxidative Stress Induces Monocyte Necrosis with Enrichment of Cell-Bound Albumin and Overexpression of Endoplasmic Reticulum and Mitochondrial Chaperones
Source: PLoS One. 2013 Mar 26;8(3):e59610. doi: 10.1371/journal.pone.0059610 (PMC3608663; doi:10.1371/journal.pone.0059610)

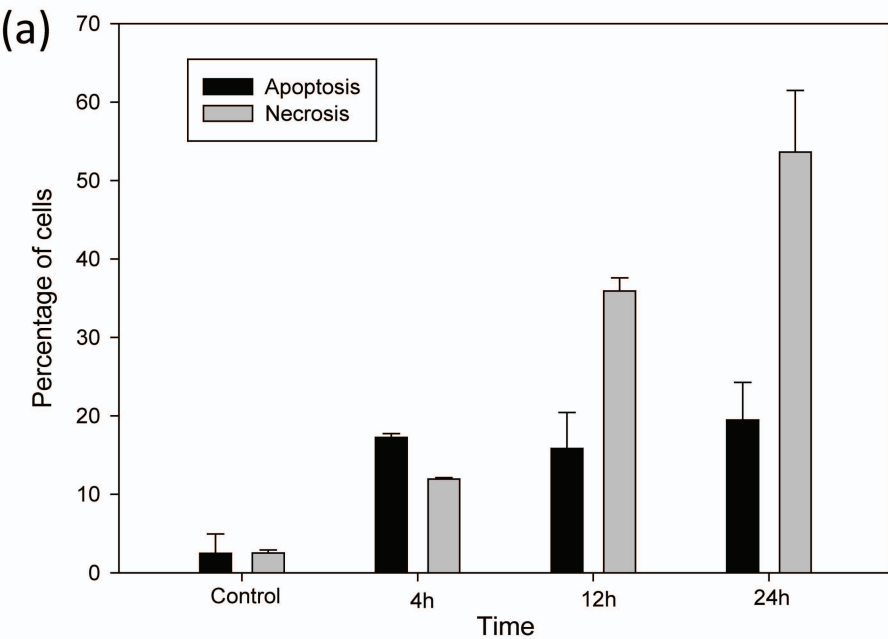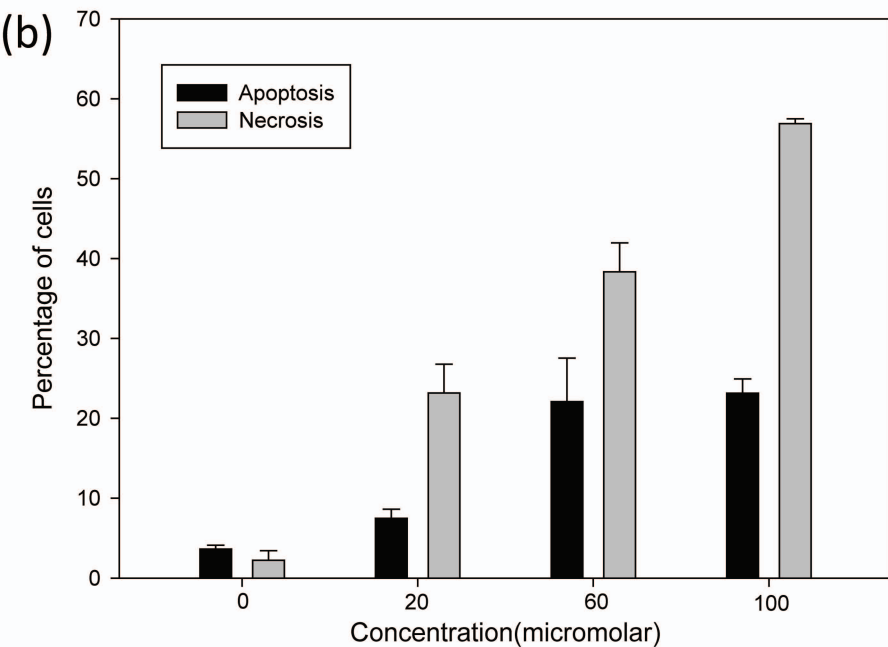

Supplement: Figure S1 — FACS analysis of azacytidine-induced cell death. THP-1 cells were treated with azacytidine at different concentrations for different periods of time. Apoptotic and necrotic cells were stained with FITC-Annexin V and Ethidium Homodimer III, respectively. (a) Percentages of apoptotic- and necrotic-cells from 100 µM azacytidine-treated cells for 0, 4, 12, and 24 h. (b) Percentages of apoptotic- and necrotic-cells from untreated, 20, 60, and 100 µM azacytidine-treated cells for 24 h. Results are expressed as the mean of three experiments. (PDF) [file pone.0059610.s001.pdf]

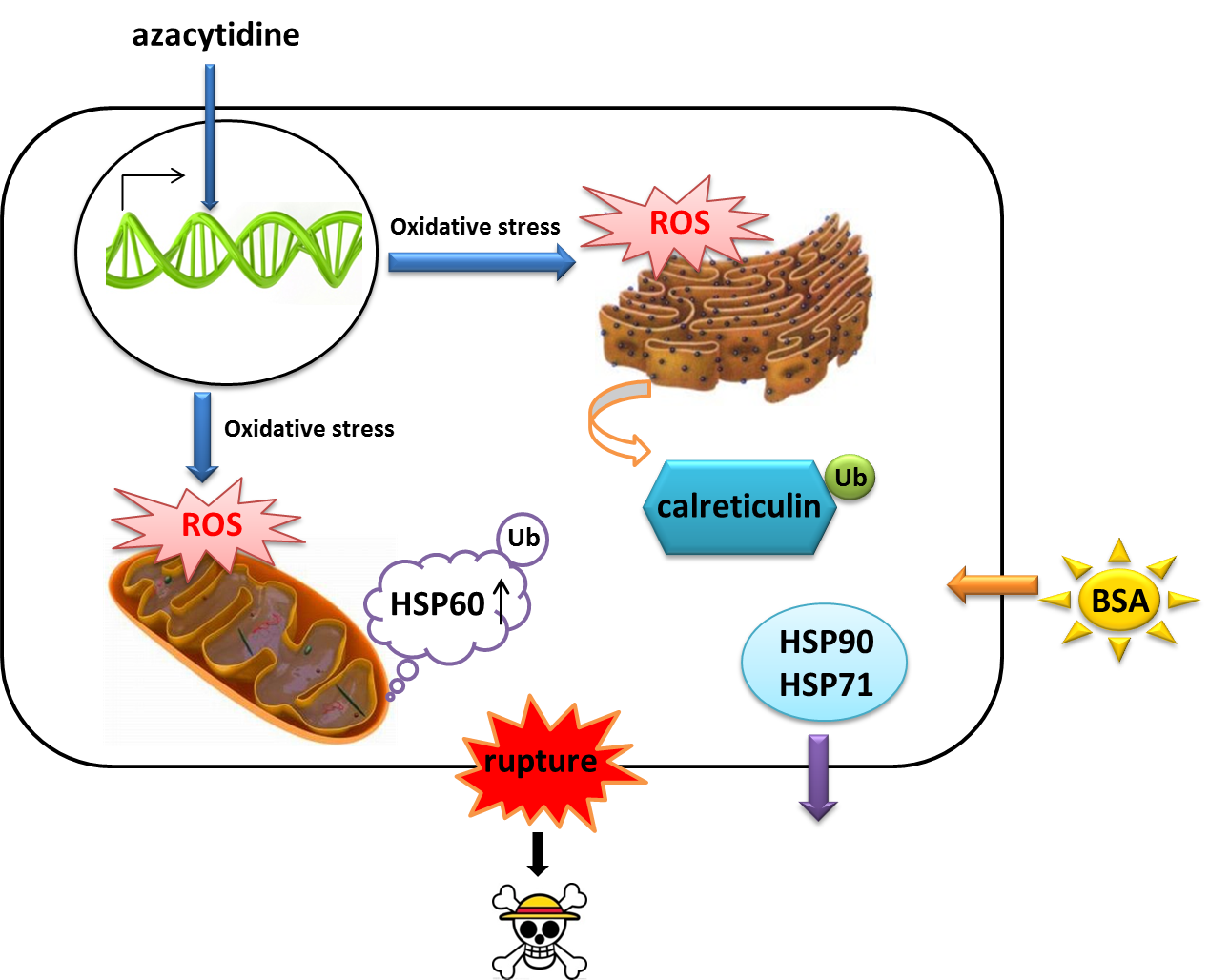

Supplement: Figure S4 — Model of azacytidine-induced changes in necrotic monocytes. (TIF) [file pone.0059610.s004.tif]
