# Supplementary material for: Oxidative Stress Induces Monocyte Necrosis with Enrichment of Cell-Bound Albumin and Overexpression of Endoplasmic Reticulum and Mitochondrial Chaperones
Source: PLoS One. 2013 Mar 26;8(3):e59610. doi: 10.1371/journal.pone.0059610 (PMC3608663; doi:10.1371/journal.pone.0059610)
Supplement: Table S1 — Primers used for qPCR analysis in this work. (DOCX) [file pone.0059610.s005.docx]

Supplementary table 1: Primers used in qPCR

| Gene | Sequence |
| --- | --- |
| HSP90AA1 forward primer | AGGAGGTTGAGACGTTCGC |
| HSP90AA1 reverse primer | AGAGTTCGATCTTGTTTGTTCGG |
| HSC71 forward primer | ACTCCAAGCTATGTCGCCTTT |
| HSC71 reverse primer | TGGCATCAAAAACTGTGTTGGT |
| 18SrRNA forward primer | GTAACCCGTTGAACCCCATT |
| 18SrRNA reverse primer | CCATCCAATCGGTAGTAGCG |
